# Sex Hormones and Inflammation Role in Oral Cancer Progression: A Molecular and Biological Point of View

**DOI:** 10.1155/2020/9587971

**Published:** 2020-06-27

**Authors:** Maria Contaldo, Mariarosaria Boccellino, Giuseppa Zannini, Antonio Romano, Antonella Sciarra, Alessandra Sacco, Giuliana Settembre, Mario Coppola, Angelina Di Carlo, Luigi D'Angelo, Francesco Inchingolo, Antonia Feola, Marina Di Domenico

**Affiliations:** ^1^Multidisciplinary Department of Medical, Surgical and Dental Specialties, University of Campania “Luigi Vanvitelli”, Naples, NA, Italy; ^2^Department of Precision Medicine, University of Campania “Luigi Vanvitelli”, Naples, NA, Italy; ^3^Department of Biology, University of Naples “Federico II”, Naples, NA, Italy; ^4^Department of Experimental Medicine, University of Campania “Luigi Vanvitelli”, Naples, NA, Italy; ^5^Cell Biology and Biotherapy Unit, Tumor National Istitute “Fondazione G. Pascale”-IRCCS, Naples, NA, Italy; ^6^Department of Medico-Surgical Sciences and Biotechnologies, “Sapienza” University of Rome, Rome, Italy; ^7^Department of Interdisciplinary Medicine, School of Medicine, University of Bari Aldo Moro, Bari, Italy; ^8^Sbarro Institute for Cancer Research and Molecular Medicine, Department of Biology, College of Science and Technology, Temple University, Philadelphia, PA, USA

## Abstract

Oral cancers have been proven to arise from precursors lesions and to be related to risk behaviour such as alcohol consumption and smoke. However, the present paper focuses on the role of chronic inflammation, related to chronical oral infections and/or altered immune responses occurring during dysimmune and autoimmune diseases, in the oral cancerogenesis. Particularly, oral candidiasis and periodontal diseases introduce a vicious circle of nonhealing and perpetuation of the inflammatory processes, thus leading toward cancer occurrence via local and systemic inflammatory modulators and via genetic and epigenetic factors.

## 1. Introduction

Oral squamous cell carcinoma (OSCC) is the most frequent malignancy of the oral cavity, accounting for more than 90% of malignant tumors of this anatomic site [1, 2] and often arise from precursor lesions [3–5].

OSCC overall survival has been proven to be strictly related to the time of diagnosis and, in addition to the classical prognostic indicators [6–8], biological markers [9–13] and the recognized risk factors, such as alcohol and smoke [14], a key role in carcinogenesis has been recently assigned to chronic inflammation and/or infections, which may lead toward genetic and epigenetic changes involved in the malignant transformation of the oral keratinocytes [15–18].

## 2. Action of Acute and Chronic Inflammations in Oral Disorders

Inflammation is an early protective and localized response of the tissue to infections, radiations (UV), injuries, and chemicals. The inflammatory pathway localizes and disrupts the pathogen, repairs the damaged tissue, and regulates the altered homeostasis. Depending on the duration, inflammation is acute, when it resolves in a few days or chronic, which does not resolve because of the persistence of pathogen or tissue injury and may lead to pathologies such as cancer [19, 20].

### 2.1. Action of Phlogistic Mediators in Cancer Development

The inflammation pathway is activated by innate immune cells that, thanks to their membrane receptors, identify and recognize pathogens and activate different response pathways through the production of phlogistic mediators [21]. Among the cells involved in the inflammation process, an important role is played by macrophages, involved during all phases of inflammation. In the first step of the inflammation, macrophages bound the tissue site and differentiate from circulating monocytes, acquiring distinct characteristics and functions in response to the specific pathogens. Thanks to their receptor, they recognize pathogens and lead to cytokines production by epithelial cells involving the activation of toll-like receptor (TLR) signaling. The cytokines and chemokines generated at the damage site activate and recruit neutrophils that have a pivotal role in the cascade, by trapping and killing the pathogens [22, 23]. Neutrophils are able to engulf, reduce to granules, and release the nuclear chromatin as neutrophil extracellular traps (NETs) (neutrophil extracellular traps in immunity and disease) [24], and to produce several cytokines and other phlogistic mediators that influence and regulate inflammation and immunity [25, 26]. When the immune system fails to vanquish the pathogen source of acute inflammation, chronic inflammation response is established. This pathological status is a further attempt of the body to free itself from the pathogenic insult, it influences several metabolic processes including cell homeostasis, inducing genomic changes, which in the long run can promote carcinogenesis [27].

**Figure 1:**
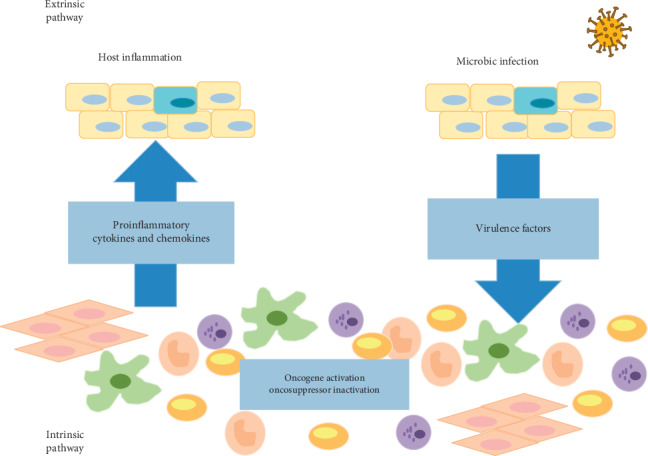
Cancerogenetic changes and inflammatory triggers are both involved in oral cancer onset by a two-way interrelated pathway, involving intrinsic and extrinsic events toward cancerogenesis. The intrinsic factors include genetic and epigenetic phenomena bringing the keratinocyte toward malignant transformation (oncogenes activation/oncosuppressor inactivation) and the production of inflammatory cancer-related mediators that recruit inflammatory cells. The extrinsic pathway is related to an underlying inflammatory/infectious state, which can promote cancerogenesis via the production of inflammatory cytokines that activate a series of transcription factors responsible for tumorigenesis. Both pathways bring toward the production of further phlogistic mediators and cancer-promoting transcription factors, thus creating a microenvironment where inflammation and cancer feed on each other.

Moreover, several studies have suggested a pivotal role of chronic inflammation in carcinogenesis and have considered it as a risk factor for most types of cancer [28–33]. According to Mantovani et al., inflammation and cancer share two pathways. The extrinsic pathway is related to those chronic inflammatory conditions that increase cancer risk; the intrinsic pathway is related to genetic alterations responsible for inflammation and tumor, such as oncogenes activation and oncosuppressor inactivation (Figure 1) [34].

Many proinflammatory mediators play a critical role in the suppression of apoptosis, proliferation, angiogenesis, invasion, and metastasis, including TNF superfamily, interleukins, chemokines, MMP-9, VEGF, COX-2 and 5-LOX [35–37].

The expression of all these proteins is mainly regulated by NF-*κ*B, a transcriptional factor that is constitutively active in most tumors and is induced by carcinogens (such as cigarette smoke), tumor promoters, and carcinogenic viral proteins. It can mediate tumorigenesis directly, acting as a growth factor for tumor cells, and indirectly through the action of its gene products [38, 39].

TNF-*α* is one of the major mediators of inflammation, is mainly produced by macrophages, and is induced by a wide range of pathogenic stimuli. Once secreted, TNF-*α* can mediate a variety of diseases, including cancer [40]: it can induce cellular transformation, proliferation, and tumor promotion [41]. TNF-*α* activates IKK, that, in turn, phosphorylates IKB, causing its rapid polyubiquitination [42, 43]. In this way, NF-*κ*B is no longer retained in the cytoplasm, can translocate to the nucleus, and promotes target genes transcription, including cIAP-1, cIAP-2, Bcl-xL, XIAP, and IEX-1L, all with antiapoptotic proprieties [44]. Moreover, NF-*κ*B suppresses the apoptotic JNK activation and mediates expression of antiapoptotic and antioxidant genes, blocking cell death and facilitating cancer cell proliferation [45]. Therefore, the balance of TNF-induced survival and death signaling is pivotal in determining the fate of cellular response induced by TNF. Modulating this balance could help to prevent cancer development [46].

Even several inflammatory interleukins are involved in tumorigeneses, such as IL-1, IL-6, IL-8, and IL-18, suggesting that inflammation is associated with cancer development. In particular, the secretion of IL-1a promotes growth in cervical carcinoma [47], while autocrine production of IL-1b promotes growth and confers chemoresistance in pancreatic carcinoma cell lines [48]. IL-6 acts as a paracrine growth factor for multiple myeloma, non-Hodgkin's lymphoma, bladder cancer, colorectal cancer, and renal cell carcinoma (RCC) [49–53]. Another important proinflammatory cytokine is IL-8, which promotes the growth and metastasis of many tumors, like astrocytomas, anaplastic astrocytomas, glioblastomas, and central nervous system cervical carcinoma metastasis [54–56].

Chemokines can be involved in cancer progression too, including angiogenesis, inflammation, cell recruitment, and migration. Therefore, they promote tumor development, inducing angiogenesis and tumor growth, directly or indirectly through the recruitment of tumor-associated macrophages [57].

### 2.2. Inflammatory Condition Involved in Oral Cancer

In the pathogenesis of oral squamous cell carcinoma are involved many oral inflammatory conditions: oral submucous fibrosis, oral lichen planus, discoid lupus erythematosus, oral ulcers related to repetitive tissue injury, and chronic periodontal disease [58].

Oral submucous fibrosis is a potentially malignant condition, characterized by dense fibrosis of the lamina propria at the affected site, by a juxtaepithelial inflammatory reaction and by atrophy or hyperplasia of the overlying epithelium. The inflammatory infiltrate comprises mainly lymphocytes, but also plasma cells and macrophages [59–62].

The process of fibrosis is characterized by the production of inflammatory mediators and growth factors, including prostaglandins, reactive oxygen species, TNF-*α*, IL-8, IL-6, TGF-*β*, platelet-derived growth factor, and basic fibroblast growth factor, by oral keratinocytes and inflammatory cells within the lamina propria [63, 64]. In the inflamed microenvironment of the submucous fibrosis TNF-*α*, IL-6 and PGE-2 may favour malignant transformation of the precancerous keratinocytes and their clonal expansion. After the acquisition of additional genetic alterations, some of these proliferating precancerous keratinocytes may obtain a malignant phenotype [65].

In addition, oral lichen planus can represent a risk for oral squamous cell carcinoma [66, 67]: it is characterized by a T cell-mediated chronic immunoinflammatory reaction against a yet undefined antigen within the basal cell layer of the oral epithelium [68]. When it happens, oral lichen planus evolves to OSCC, and the local inflammatory microenvironment provides the activation of transcriptional factors that promote the proliferation of the epithelial cells affected by the lichen planus, but also angiogenesis, invasion, and metastasis [69, 70].

Moreover, the bacteria of dental-gingival plaques and their products, through local inflammatory responses, the production of nitrosamines, and mutagenic agents formed, have the capacity to start mitogenic and antiapoptotic pathways in oral keratinocytes. Thus, oral bacteria and inflammatory mediators associated with periodontal disease may be cofactors in the promotion of oral squamous cell carcinoma [71, 72].

Therefore, there are several pieces of evidence that demonstrate the link between chronic inflammation and cancer, and inflammatory biomarkers described can be used to monitor the progression of the disease.

## 3. Role of Sex Hormones in Cancer

Hormones are naturally produced in our body and they are fundamental to guide its functions [73]. Their production is finely regulated by the human organism because even a small alteration in their quantities can be the cause of serious repercussions on the whole organism [74]. Among the many functions performed by hormones, some concern also cells proliferation and can influence the risk of cancer [75–78]. As an example, excessive levels of sex hormones (like estrogens and androgens) can promote the development of breast and prostate cancer in women and men, respectively [79, 80]. The association of the estradiol receptor (ER) with Src triggered by steroid agonists growth factors controls breast and prostate cancer cell growth. To understand this mechanism, Varricchio et al. have shown that this association is abolished in whole cells and in vitro by a six-amino-acid peptide that mimics the sequence around the phosphotyrosine residue in position 537 of the human ER-alpha, indicating that the peptide specifically targets the ER-associated Src [81].

The main mechanisms by which hormones influence cancer development are by controlling the rate of cell division, the differentiation of cells, and the number of susceptible cells [82]. Sex hormones, for example, have very marked effects on cell division in the endometrium; particularly, estrogens stimulate mitosis, while progestins oppose this effect [83, 84]. Nowadays, it is clear the mechanisms through which estrogens cause cancer [85]. It is well known that estrogen receptor ER*α* is certainly involved in cell cycle process, cell migration, and invasion (as demonstrated on MCF-7 cellular model [86]) and that there is a strong association of estrogen dose and length of exposure with increased breast cancer risk [87]. In many steroid‐dependent cancers, there is an interaction between growth factor and steroid signaling, which converge in the PI3K/AKT pathway [88, 89]. There are many experimental pieces of evidence showing that the phosphorylation of p85*α* SH3 domain, the regulatory subunit of PI3K, represents a crucial step for the activation of this pathway [90]. PI3K/AKT pathway controls cell cycle progression, cell growth, proliferation, apoptosis, adhesion, and migration through various downstream effectors and, in the end, could be involved in the growth of cancers [91]. PI3K/AKT is the most frequently mutated pathways in cancer, and most of the p85*α*^PI3K^ mutations cluster in the inter-SH2 (iSH2) domain of the molecule, which interacts with the catalytic subunit p110*α*^PI3K^ [92]. It is known that PKA phosphorylation of serine at codon 83 (S83), adjacent to N-terminus SH3 domain in the PI3K regulatory subunit p85*α*, is involved in cell cycle progression and cell survival in normal epithelium, influencing the ability of SH3 domain to interact with different partners [93, 94]. Among these, PI3K activates AKT, localizing it in the plasma membrane [95]; in turn, AKT, once activated, can have several downstream effects such as activating CREB [96], inhibiting p27, and activating mTOR [97]. The result of AKT activation is represented by the induction of cell growth and resistance to apoptosis, both processes involved in many cancers [98]. Nearly all receptors binding p85*α*^PI3K^ can cooperate with cAMP-PKA signals via phosphorylation of p85*α*^PI3K^Ser83 [88, 99]: ER*α* and retinoic acid receptor-alpha (RAR*α*) have been extensively studied for their association with PI3K and their involvement in cancer risk [100, 101]. The PI3K/AKT/mTOR pathway is altered in around 30.5% of HNSCC patients [102, 103]. The AKT/mTOR pathway plays an essential role in regulating the formation of blood vessels in both normal and cancer tissues. Angiogenesis is characterized by the secretion of vascular endothelial growth factor (VEGF), basic fibroblast growth factor (b-FGF), and interleukins such as IL-8 by cancer cells [104].

### 3.1. Sex Hormones in Oral Cancer Development and Progression

The role of sex hormones is well known in cases of prostate, breast, and endometrial carcinomas; instead, the involvement of sex hormones in HNC (head and neck cancer) is still controversial. Probably sex hormones affect cancer risk by increasing the number and mitotic rate of the epithelial cells in the organ concerned [105]. In fact, high mitotic rates can increase cancer risk because there are more chances of mutations that can be replicated before being repaired and can also increase the growth of early tumors. Moreover, in the case of estrogens, some metabolites of estradiol may cause mutations by directly damaging the DNA, but the importance of this possible process has not been established yet [106]. HNC includes all malignant tumors that derive from the moist squamous cell mucosa or lining of the head and neck regions and comprises malignancies of the nasal cavity and paranasal sinuses, oral cavity, pharynx, larynx, and salivary glands [107]. Recent literature suggests that, apart from the major established risk factors (alcohol and smoking), female sex hormones may contribute to head and neck carcinogenesis, and it indicates certain endocrine involvement in its development [108].

The expression of sexual hormone receptors in some tumors underlines a role for these molecules in cancer pathogenesis. Colella et al. investigated the presence and expression levels of sexual steroid receptors in OSCC. They found that ER*α* mRNA is more expressed in the OSCC than in control tissues, while opposite results have been obtained for AR [107].

It is known that AR binds to specific DNA sequences and affects the transcription of various genes. Fan and Malhelm suggested that AR may have a role in the pathogenesis of salivary duct carcinoma through the production of an epidermal growth factor receptor and making growth factor-*α* autocrine pathway similar to what happens in prostatic carcinoma [109]. In addition, some studies have shown that androgens affect the expression of proto-oncogenes (like c-myc) and apoptotic factors (like the bcl-2 family) in lacrimal, salivary, and prostatic tissues of both mice and rats, as well as in cell line models [110]. Therefore, the expression of sex hormone receptors in some tumors certainly suggests a role for these receptors in cancer pathogenesis, progression, and therapy.

Some studies have demonstrated that elevated prolactin levels in HNC can be a marker of poor prognosis [111]. Few other studies have shown increased levels of FSH, LH, and prolactin and decreased ratio of testosterone/estradiol in tongue cancer patients. This indicates a disruption of the pituitary-adrenal-testicular axis and suggests that these hormones might play a crucial role in the development and progression of oral cancer [112].

In addition, some recent reports have shown pieces of evidence that salivary gland tumors are similar to breast cancer from a molecular and cellular point of view [113]. Both tumors show a similar expression of progesterone associated with cancer progression [114]. Moreover, estrogens may induce the movement of precancerous cells in the mouth and promote the spread of head and neck cancers [115].

## 4. Functions of Infection and Epigenetic Modification in Oral Carcinogenesis

Hence, chronic infections and/or dysimmune diseases may sustain a chronic inflammatory state, which represents a risk factor for carcinogenesis. Indeed, immunity and inflammation usually fight against the pathogens to reduce the risk of dissemination and to support repairing mechanisms. However, in some cases, these systems are unable to totally eradicate the infection and/or its recurrence, thus supporting genetic and epigenetic changes toward carcinogenesis in the cells of the involved tissues. Furthermore, in the tissues where the inflammatory/immune responses take place [116], the onset of oxidative stress, associated with the chronic inflammatory state, takes part in the pathogenic mechanisms [117–119] responsible for carcinogenesis of such oral diseases [120–122]. Different chronic inflammatory and dysimmune conditions may affect the oral structures [123, 124], thus giving rise to an endless circle of inflammatory events leading toward chronicity and increase of the risk of cancer transformation and metastasis [125]. This condition is a significant attributable factor for poor prognosis and circulating tumor cells CTCs are shedding by a primary clone and are responsible for oral cancer-related deaths [126]. Various pilot studies show that serum miRNA signature might be a potential biomarker for early detection of cancer and, in particular, a three-plasma miRNA panel (miR-222-3p, miR-150-5p, and miR-423-5p) may be useful to monitor malignant progression from OSCC [127, 128]. miRNAs are considered biomarkers for gene expression. They influence the regulation of cellular processes such as cell differentiation, proliferation, and apoptosis. The aberrations in miRNA expression predict that miRNA identification in oncogenesis might reveal networks or targets that are suitable for cancer diagnostic markers or cancer therapy [129–131].

### 4.1. Oral Infections Involvement in Inflammation Process

Not only the chronic inflammation infection-related is responsible for cancerogenesis, but also bacteria, fungi, and viruses themselves are strongly implicated as etiological factors in cancers [132]. The most investigated associations are between HPV and oropharyngeal carcinomas and *Candida* spp overinfected oral lichen planus and OSCC [133–135].

Oral microbiota homeostasis is fundamental to maintain the state of oral health. Each dysbiosis or unbalanced spread of pathogens give rise to several infectious diseases of both teeth and mucosae, which can perseverate in a chronic condition [136], during which the reparative processes fail, due to the inflammatory effects themselves. As reported by Zhang et al., OSCC tissues show a higher number of bacteria and significant changes in microbiota compared to the normal tissues in the buccal mucosa [137]. Furthermore, oral microbiota unbalance can influence not only oral cancer onset, but it can also lead to systemic diseases and chronic inflammation, such as cardiovascular diseases, diabetes, rheumatoid arthritis, neurodegenerative diseases, and skin psoriasis [138]. The most investigated oral disease where infection, oral dysbiosis, chronic inflammation, and systemic involvements have been variously proven is periodontitis, a chronic inflammatory disorder responsible for the loss of dental elements. Periodontal chronic inflammation is triggered by specific bacterial species colonizing the gingival pockets of predisposed subjects and sustained by genetic [139, 140] and environmental factors [141]. Eke et al., in 2012, evaluated the prevalence of the periodontal disease in the United States, providing evidence for the priority function of transforming growth factor-*β* platelet-derived growth factor, IL-1 and keratinocyte growth factor, which may be related the crosstalk between fibroblasts and keratinocytes, affecting wound and periodontal healing process [142]. If the association among periodontitis and endocarditis has been proven by several studies [143], the interrelationship among periodontitis associated bacteria and cytokine expressions, and their responsibilities toward the onset of bone and inflammatory systemic diseases, such as osteoporosis and rheumatoid arthritis, have also been elucidated by Ballini et al. in 2015 [144], which reported that bacterial-associated molecules, such *Porphyromonas gingivalis*' ones, may interact with their surface receptors in the immune cells, thus inducing the production of several cytokines and chemokines with a proinflammatory role and allowing the activation of mechanisms related to osteoporosis and rheumatoid arthritis. Among common infections affecting the oral cavity, Candidiasis often arises in subjects suffering systemic diseases [145] and/or local factor [146], or salivary alterations [147] which dysregulate the oral microbiome, leading to the spread of a series of microbial and fungal infections [148–150], which may persist in a chronic or recurrent state [151], and that can be associated with the oral mucosae cancerization. The expression and phosphorylation state of EGFR and the expression of cadherins could be considered to monitor oral cancer progression.

### 4.2. Relationship between Genetic and Epigenetic Changes in Cancer

**Figure 2:**
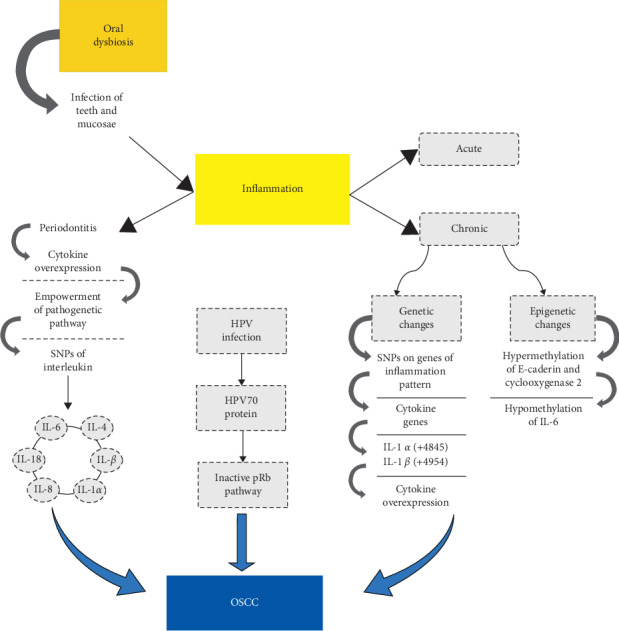
Schematic representation of the relation between oral squamous cell carcinoma (OSCC), inflammation, and periodontitis. The figure represents the different mechanisms involved in the development of the OSCC in relation to inflammation. The chronic one is correlated to periodontitis disease and may lead to genetic and epigenetic changes like cytokine, SNPs. This mutation involved mainly IL-*α* and IL-*β* which are overexpressed.

Genetic research has underlined several interactions between environmental and epigenetic factors, aiming to highlight the molecular genetic markers with clinical signs and risk factors for periodontitis [152]. A more modern scientific approach to understand periodontitis vulnerability is involved in recognizing the susceptibility to inflammatory process for DNA mutations of specific cytokine genes. While the prognostic significance of the quantitative and topographic dysregulation of proteins of the cadherin families and the methylation of their genes or downregulation of their mRNA have been widely proven to be related to cancer progression [153–157], authors such as Loo et al. hypothesize their responsibilities toward chronic periodontitis too [158]. Many investigators have evaluated the association between genetic polymorphisms in humans with different periodontal disease patterns; these studies pointed out that genetic polymorphisms in some candidate genes are involved in interindividual variations in their response to periodontitis [159]. Genetic bases, in fact, are involved in the proinflammatory signaling pathways, through proteins expression and regulation [160]: changes in gene expression seem to generate inflammatory cytokines activation, responsible for periodontitis pathogenetic cascade, as recently assessed by Rudick et al. [161]. The genetic model predisposition for early-onset periodontitis was hypothesizes in 1994 by Marazita et al.: they focused on individual's immune response as the key role to interpret the susceptibility to progression of periodontal disease. Polymorphonuclear leukocytes decrease, in fact, is noted to strongly affect vulnerability to periodontitis. To date, moreover, it seems to be established that cytokines overexpression, influenced by the immune cells ongoing, leads to empowerment of the pathogenetic pathway. Several kinds of cytokines seem to be involved: SNPs of IL-1*α*, IL-1*β*, IL-4, IL-6, IL-8, and IL-18 located in different regions of the cytokine genes have been shown to affect the risk of the disease in several populations [162]. The most suggestive aspect is related to IL-1 polymorphisms, which has shown as a promoter in the enhancement of periodontal disease. Indeed, in 1995 Hassel and Harris have paid great attention to “Periodontitis associated genotype (PAG),” as a significant increasing marker in patients with advanced periodontitis and dental caries [163]. Epigenetic linkage with periodontal disease seems to recognize not only IL-1 polymorphisms relationship, but also a moderate association with hypermethylation of E-Cadherin and cyclooxygenase 2 [164, 165]. Recently, Cantore et al. confirmed these data by molecular analyses reporting a statistically significant correlation between the severe form of periodontitis and the presence of IL-1*α* (+4845) and IL-1*β* (+3954) single nucleotide polymorphisms (SNPs) [166], refuting previous studies that identified a marked contribution of IL-1 in development of periodontitis and that refused it as essential on disease risk [167]. In addition, Gomez et al. in 2009 found hypomethylation of IL-6 gene in people with periodontal disease [168] and Küchler et al., recently, have deeply reviewed the literature to investigate how apical periodontitis pathogenesis ranges from microbial to genetic factors [137]: the most suggestive results emphasized the interindividual variation in apical periodontitis pathogenesis and severity of host response (phenotype). Although literature showed a lack of studies in the field of epigenetic factors related to periodontitis, a strong association has been found between IL-1B and TNF-*α* polymorphism and persistent apical periodontitis [169]. Further studies aiming to rate the genetic risk for periodontitis have focused on molecular levels of IL-1, IL-6, TNF-*α*, Vitamin-D receptor, Fc-gamma receptor, IL-10, and matrix metalloproteinase [170, 171]. The interest in the determination of the diagnostic value of metalloproteinases, in fact, is well known not only in periodontal diseases [172] but also in caries, pulp, and periapical inflammations [173, 174] and some cancers [175–177]. Moreover, other authors focused on genetic disorders related to periodontitis, since several syndromes, such as Chèdiak–Higashi, trisomy 21, Ehlers-Danlos, Papillon-Lefèvre, Haim-Munk, leukocyte adhesion deficiency (LAD), and lazy leukocyte syndrome, show a periodontal disease history, due to anomaly in aggregation and action of neutrophils [178]. Although there is still no evidence about an interlink between syndromic genetic mutations and periodontitis and no genetic tests are currently utilized, the Research Science and Therapy Committee of American Academy of Periodontology in 2005 has compiled an informational paper showing promising results of tests for genetic IL-1 polymorphisms as a genomic risk factor for periodontitis [179]. Recently, it is demonstrated that laser irradiation enhanced mitogen activity compared to nonlasered control cells on the growth rate and differentiation of human osteoblast-like cells seeded on titanium or zirconia surfaces [180]. Furthermore, a recent systematic review elucidated a statistically significant difference in the levels of some salivary cytokines (IL-6, CCL3, TGF-*β*, CXCL8, GM-CSF, and TNF-*α*) between subjects with denture stomatitis and controls (*P* < 0.05) and, on the other side, the lack of significant differences in the same group/control pair, as regards IL-2, IL-12, IFN-*γ*, IL-4, IL-8, IL-10, IL-17, TNF-*α*, and ICAM-1 [181]. Significant differences in levels of some cytokines have also been identified in dental inflamed pulp [182], where a significant increase in levels of IL-1*β*, IL-2, IL-6, IL-8, and TNF-*α* was typically found in irreversible pulpitis samples, in comparison to normal pulp samples (Figure 2). Last but not least, the role of some kinds of viral infections [183] is the most reported condition of carcinogenesis of the head and neck mucosae [184–186], particularly with regard to oropharyngeal cancers [18] but also to OSCCs, where Pannone et al. identified among a series of OSCCs, a subgroup characterized by HPV infection (10.5%), in which the oncogenic role of HPV70 was confirmed, via p53 protein inactivation, and the consequent cell immortalization promotion and the oncogenic risk of HPV-53 which produced HPV-E7 protein and inactivated pRb oncosuppressor pathway [187–190].

## 5. Conclusions

The present work aimed to highlight the roles of sex hormones, oral chronic infections, and auto/dysimmune diseases in the onset of oral cancerization. The responsibilities of estrogens and androgens have been clearly proven about breast and prostate cancers, while estrogen receptor-alpha (ER*α*) and retinoic acid receptor-alpha (RAR*α*) have been extensively studied for their involvement in cancer risk, also with regard to oral cancers. Many pieces of evidence reported in this review may help to understand the risk factors that cause HNC in addition to the traditional risk factors, that is, tobacco and alcohol exposure. Here, we discuss that sex hormones may influence the normal-dysplasia-cancer evolution of the oral mucosa, and this could contribute to understand the molecular pathways of chemotherapy for OSCC. In fact, we think that it could be useful to consider specific antagonists against sex hormone receptors. Even treatments such as vitamin A and beta carotene have been reported to be effective in healing oral leukoplakia [191] and RAR*β* expression inhibits oral squamous cell carcinoma bone invasion [192]. Furthermore, chronic inflammation occurring during oral infections and/or dysimmune diseases represents a risk factor for carcinogenesis, as well reported with regard to the oncogenic potential of such oral infections sustained by *Candida* spp. Lastly, in addition to the consolidate association among periodontitis and heart diseases such as endocarditis [193], an increasing number of researches point on the role of *Porphyromonas gingivalis*, usually reported as involved in the onset of periodontitis, as associated with oral and extraoral carcinogenesis [194]. The present report suggests that the described signaling proteomic and cellular effectors may have therapeutic applications for oral squamous patients [195–198]. In conclusion, inflammatory and infectious processes sustaining periodontitis and other oral affections are associated with the onset of oral cancer, sustained by local and systemic inflammatory cascades and by genetic and epigenetic factors.

## Abbreviations

5-LOX: 5-lipoxygenase
AR: Androgen receptor
b-FGF: Basic fibroblast growth factor
Bcl-xL: B-cell lymphoma-extra large
cAMP: Cyclic adenosine monophosphate
CCL3: Chemokine (C-C motif) ligand 3
cIAP-1, -2: Cellular inhibitor of apoptosis protein-1, -2
COX-2: Cyclooxygenase 2
CREB: cAMP response element-binding protein
CXCL8: Interleukin 8
ER*α*: Estrogen receptor-alpha
FSH: Follicle-stimulating hormone
GM-CSF: Granulocyte-macrophage colony-stimulating factor
HNC: Head and neck cancer
HNSCC: Head and neck squamous cell carcinoma
HPV: Human papilloma virus
ICAM-1: Intercellular adhesion molecule 1
IEX-1L: Radiation-inducible immediate-early gene
IFN-*γ*: Interferon gamma
IKK: I*κ*B kinase
IL-: Interleukin
LAD: Leukocyte adhesion deficiency
LH: Luteinizing hormone
miR: microRNA
MMP-9: Matrix metallopeptidase 9
mTOR: Mammalian target of rapamycin
NETs: Neutrophil extracellular traps
NF-*κ*B: Nuclear factor kappa-light-chain-enhancer of activated B cells
OSCC: Oral squamous cell carcinoma
p110*α*^PI3K^: Phosphoinositide-3-kinase p110 alpha catalytic subunit
p85*α*^PI3K^: Phosphoinositide-3-kinase p85 alpha regulatory subunit
PAG: Periodontitis associated genotype
PGE-2: Prostaglandin E2
PI3K/AKT: Phosphoinositide-3-kinase/Protein kinase B
PKA: Protein kinase A
RAR*α*: Retinoic acid receptor-alpha
SH2 and 3: Src H2 homology domain
SNPs: Single nucleotide polymorphisms
Src: Proto-oncogene tyrosine-protein kinase
TGF-*β*: Transforming growth factor beta
TLR: Toll-like receptor
TNF-*α*: Tumor necrosis factor alpha
VEGF: Vascular endothelial growth factor
XIAP: X-linked inhibitor of apoptosis protein.
